# Ultrasensitive detection of saccharides using terahertz sensor based on metallic nano-slits

**DOI:** 10.1038/s41598-020-60732-w

**Published:** 2020-02-28

**Authors:** Jianyuan Qin, Wei Cheng, Baojuan Han, Yong Du, Zhanghua Han, Zongshan Zhao

**Affiliations:** 1 Center for Terahertz Research, China Jiliang University, Hangzhou, 310018 China; 2 Advanced Launching Co-innovation Center, Nanjing University of Science and Technology, Nanjing, 210094 China; 3 College of Environmental Science and Engineering, Qingdao University, Qingdao, 266071 China

**Keywords:** Metamaterials, Terahertz optics

## Abstract

Unambiguous identification of trace amounts of biochemical molecules in a complex background using terahertz spectroscopy is extremely challenging owing to the extremely small absorption cross sections of these molecules in the terahertz regime. Herein, we numerically propose a terahertz nonresonant nano-slits structure that serves as a powerful sensor. The structure exhibits strongly enhanced electric field in the slits (five orders of magnitude), as well as high transmittance over an extra-wide frequency range that covers the characteristic frequencies of most molecules. Fingerprint features of lactose and maltose are clearly detected using this slits structure, indicating that this structure can be used to identify different saccharides without changing its geometrical parameters. The absorption signal strengths of lactose and maltose with a thickness of 200 nm are strongly enhanced by factors of 52.5 and 33.4, respectively. This structure is very sensitive to thin thickness and is suitable for the detection of trace sample, and the lactose thickness can be predicted on the basis of absorption signal strength when the thickness is less than 250 nm. The detection of a mixture of lactose and maltose indicates that this structure can also achieve multi-sensing which is very difficult to realize by using the resonant structures.

## Introduction

The demand for new chemical and biological sensing methods operating in the optical, infrared, terahertz (THz), and microwave regimes has been dramatically increasing. In particular, inter- and intramolecular vibrations of biochemical molecules occur in the THz regime, and thus these molecules exhibit unique fingerprint features in this regime. Consequently, THz technology has unique advantages in biochemical sensing^1,2^. The emergence of various artificial materials raises the possibility of highly sensitive molecular fingerprint detection using THz spectroscopy^3,4^. In recent years, many works in this field have been dedicated to enhancing the sensitivity of THz sensing. Several strategies based on artificial materials have been proposed in an effort to attain higher sensitivity during THz sensing, including parallel-plate waveguides^5^, split-ring resonators^6^, and photonic crystals^7^. The working principle of these approaches is to make the resonance frequency of sensor structure at a desired position that matches the analyte’s characteristic frequency by adjusting its geometrical parameters. Therefore, the geometrical parameters of the structure must typically be redesigned for the detection of different analytes.

Subwavelength metallic structures, in the form of slits, can focus an enhanced electric field inside the apertures and achieve extraordinary optical transmission (EOT)^8,9^. The emergence of EOT in these subwavelength apertures is generally attributed to surface plasmon resonances^10^. A slit with a high aspect ratio has a strong field enhancement within it, which can be applied to sensitive detection. A single nano-slit was utilized to detect an extremely small quantity (approximately 40 ng) of 1,3,5-trinitroperhydro-1,3,5-triazine (RDX)^11^. A dramatic change in the transmittance was observed at the characteristic frequency of RDX and the deduced molecular absorption enhancement reached a value of 8 × 10^6^. Two-dimensional periodic nano-slits were exploited to detect carbohydrate molecules^12^. The results demonstrated that this structure could be used to distinguish various carbohydrate molecules by observing the resonance changes in transmittance even at low concentrations. Furthermore, by redesigning the geometrical parameters of the nano-slits structure, several types of avian influenza viruses could be categorized according to the resulting resonant frequency shift and mass-normalized transmittance change^13^. An accurate control method of THz transmission by tuning filling factors of Au nanoparticles inside nano slot antennas was also reported^14^. The comparison of THz signals between the sweeping and random dropping manifested that the sweeping was far sensitive with more than about 3.5 times transmittance reduction as compared to the other, providing important clues in reinterpretation of sample deposition for further demands in developing THz molecule sensors. Although the detection sensitivity was satisfactory in these structures mentioned above, they cannot realize the detection of different analytes without redesigning their geometrical parameters due to their resonant characteristics. In these approaches, the structure was designed to possess a resonance that matched the characteristic resonance of the analyte. Upon changing the analyte, the geometrical parameters of the structure must therefore be redesigned to realize resonance matching. Recently, light transmission through a single subwavelength slit in a thin perfect electric conductor screen was analytically described, and it was found that the slit could afford a field enhancement of ~10^2^ and a transmittance efficiency of ~10 in the infrared and THz regimes, while the associated array of slits would display nonresonant perfect transmission^15^. The contrast analysis shows that the nonresonant structures can support relatively high transmittance over a wide frequency range, but weaker filed enhancement compared with the resonant structures. As a result, sensor based on nonresonant structures can realize the detection of different analytes without redesigning their geometrical parameters because of the wide working frequency band, however, its sensitivity is limited due to the weak filed enhancement. Thus, it is necessary to improve the filed enhancement in the nonresonant structures to make them more applicable to sensing detection.

Aiming to the features of the nonresonant structures, we herein numerically propose a method of ultrasensitive detection of different analytes based on a metallic nano-slits structure without redesigning its geometrical parameters. The electric field in the slits is enhanced by five orders of magnitude. For the sensing performance of this metallic nano-slits structure, the absorption signal strength of lactose is strongly enhanced by a factor of 52.5. The relation curve between the absorption signal strength and the lactose thickness variation indicates that the structure is very sensitive to thin thickness and is applicable to the detection of trace sample. The lactose thickness can be predicted based on the absorption signal strength when the thickness is less than 250 nm. What’s more, this metallic nano-slits structure can also be applied to the fingerprint detection of maltose without redesigning its geometrical parameters.

## Structure and Model

**Figure 1:**
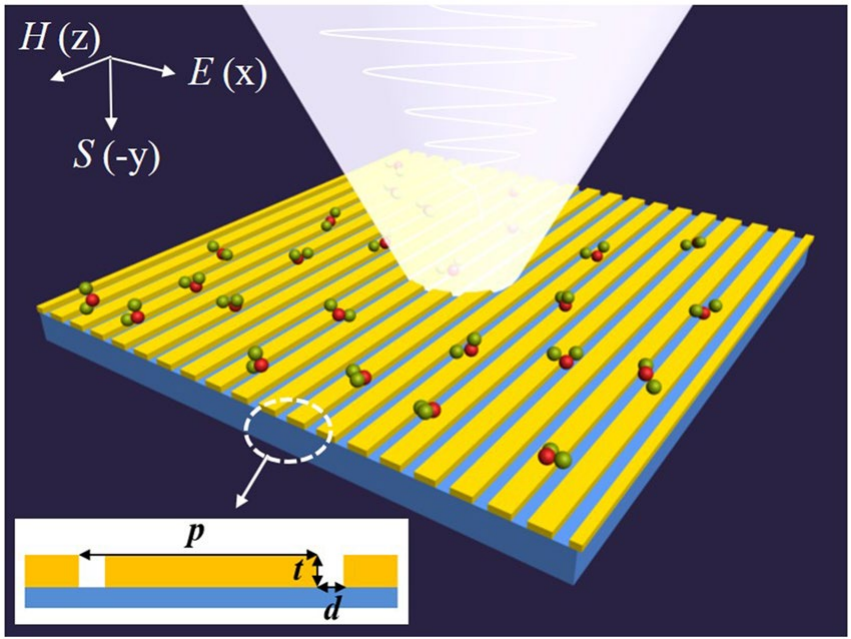
Schematic diagram of sensing using metallic nano-slits. The inset shows the cross section of the metallic nano-slits, which are defined by the width *d*, thickness *t*, and periodicity *p*.

A schematic diagram of the proposed metallic nano-slits structure is presented in Fig. 1. The structure is composed of an array of gold nano-slits on a quartz sheet. The unit cell of this considered configuration consists of a gold nano-slit on the surface of a 500 μm thick quartz sheet with a refractive index of 2. The slit possesses a width of *d* = 100 nm, a height of *t* = 50 nm (the same thing as gold film thickness), and a periodicity of *p* = 10 μm in the x axis (as shown in the inset of Fig. 1). It is important to note that the assumption of gold as a perfect conductor as normally used for THz metamaterials is not applied here^16^. This is because the skin depth of gold at 0.1 THz is 250 nm^16,17^ that is clearly larger than the nano-slit parameters (film thickness *t* = 50 nm and width *d* = 100 nm). For a better understanding of this nano-slits structure, Finite element analysis for this structure is carried out. The THz wave perpendicularly strikes the surface of metallic nano-slits along the y axis. The input and output ports in the y axis are periodic. Drude model is adopted for the calculation of the dielectric parameters of gold^18^:

1 $$\varepsilon (\omega )={\varepsilon }_{\infty }-\frac{{\omega }_{{\rm{p}}}^{2}}{\omega (\omega +i{\rm{\gamma }})}$$

where *ε*_∞_ = 1, plasma frequency *ω*_*p*_ = 1.7 × 10^4^ THz, and decay frequency γ = 40.7 THz for gold.

Fingerprint detections of lactose and maltose are investigated to study the sensing characteristics of this metallic nano-slits structure. These two saccharides are chosen because they exhibit strong vibrational resonances at 0.529 THz^19^ and 1.12 THz^20^, respectively, and they are both located in the working frequency band of the metallic nano-slits. The working frequency band here refers to the region that exhibits strong electric field enhancement and high transmittance. Lactose is characterized using the Lorentz model with parameters referred to the data in the previous report^21^, while maltose is characterized using the Lorentz model with parameters extracted by fitting the Lorentz model to experimental data derived from a public THz spectroscopic database (Terahertz Database, RIKEN)^22^.

## Result and Discussion

**Figure 2:**
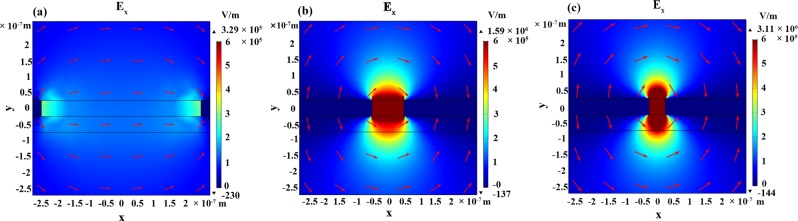
Simulated horizontal electric field distribution around a slit width a width of 500 nm (**a**), 100 nm (**b**), and 50 nm (**c**) at 0.529 THz. The red arrows illustrate the direction of the electric field.

Fig. 2 shows the horizontal electric field distribution around a slit with a width of 500 nm (a), 100 nm (b), and 50 nm (c) at 0.529 THz. The red arrows illustrate the electric field direction. In all case, the electric field is completely concentrated around the slit without penetrating into the metal. This is because the electric field around the slit is normal to the slit wall, at which point it is terminated by surface charges^16^. It also shows a smaller slit width leads to a stronger electric field (five orders of magnitude) around the slit, which agrees well with the previous reports^23,24^. According to a previous report^25^, the enhancement factor (EF) is used to decide the performance of our metallic nano-slits structures with different slit width. The EF is defined as (I_EF_/I_0_) × (A_0_/A_EF_), where I_EF_ is the enhanced signal intensity, I_0_ is the unenhanced signal strength, A_EF_ represents the area covered with analytes that contributes to signal enhancement with nano-slits structure, and A_0_ denotes the area of analytes on a reference substrate. When the 100 nm lactose layer is deposited on quartz sheet, the area covered with lactose A_0_ in one period is 1 μm^2^ (10 μm * 100 nm = 1 μm^2^, where 10 μm is the structure’s period), and the calculated unenhanced signal strength I_0_ is 0.12%. In the case of metallic nano-slits structure with 500 nm slit width, the area covered with lactose A_EF_ in one period is 5 × 10^−2^ μm^2^ (including the area inside the nano-slit and the area of fringe field over the nano-slit), and the calculated enhanced signal strength I_EF_ is 2.0%. Therefore, the corresponding EF in this case is estimated as 333. Similarly, the EF of nano-slits structure with 100 nm slit width is 1.1 × 10^4^, while the EF of nano-slits structure with 50 nm slit width reaches up to 3.1 × 10^4^. Such a high EF value will effectively increase the absorption cross section of analytes and permit their highly sensitive detection.

**Figure 3:**
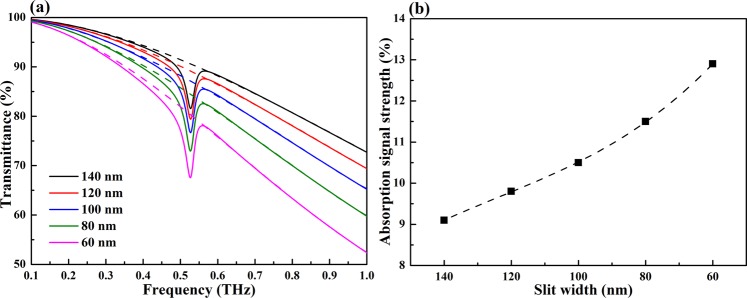
(**a**) Transmittance spectra of lactose on the metallic nano-slits structure with a slit width of 140 nm, 120 nm, 100 nm, 80 nm, and 60 nm. The dashed lines represent the fitting lines. (**b**) The absorption signal strength as a function of slit width.

To further study the effect of slit width on the absorption signal strength of analyte, the slit width of the metallic nano-slits is changed from 140 nm to 60 nm with an interval of 20 nm. The absorption signal strength is defined as the difference between the fitting line (dashed line) and transmittance line at the characteristic frequency of the analyte. Here lactose is selected as a representative analyte. The thickness of lactose layer coating on the slits is set to 200 nm. During the coating of the nano-slits structure surface with analyte, the slits fill up initially followed by covering of the gold surface. Therefore, the analyte thickness throughout our study refers to the thickness from the bottom of the slits to the top. The results are shown in Fig. 3, the black dashed line in Fig. 3(b) represents a polynomial fitting describing the relationship between the absorption signal strength and the slit width. It is found that as the slit width decreases, the absorption signal strength of lactose increases obviously (Fig. 3(a)), ranging from 9.1% to 12.9% (Fig. 3(b)). This is due to the fact that as the slit width decreases, the electric field enhancement becomes much stronger (Fig. 2) and thus the absorption signal strength enhances as expected. It is worth reminding that as the slit width decreases, the rate of decline in transmittance accelerates with the frequency increasing. That is, the working frequency band of this structure reduces as the slit width decreases although a higher absorption signal strength of analyte is achieved in this case. Therefore, the choice of slit width should be balanced between its working frequency band and the absorption signal strength of analyte. In the following study, a slit width of 100 nm is used.

**Figure 4:**
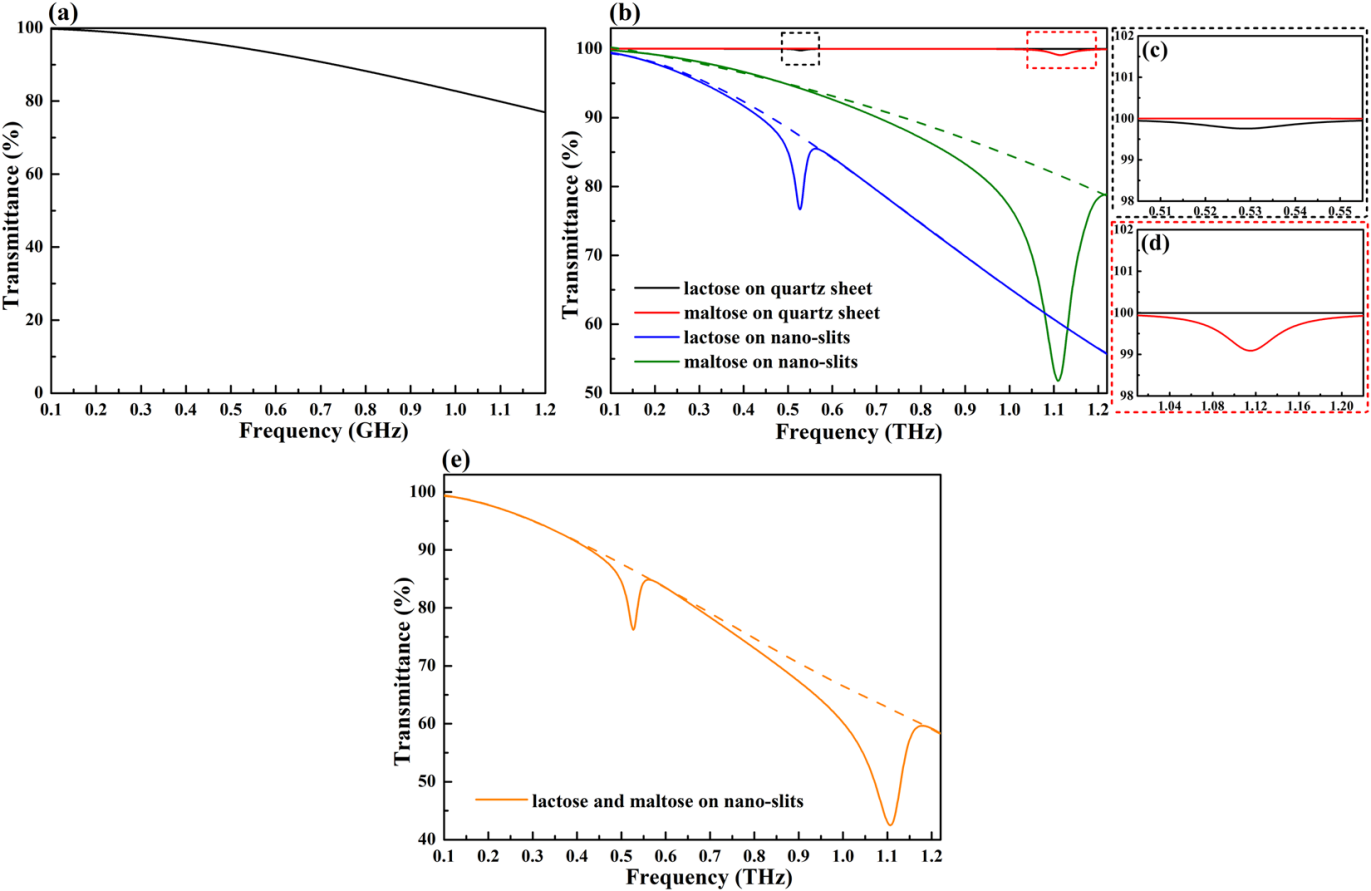
(**a**) Transmittance spectrum of the metallic nano-slits structure in the working frequency range. (**b**) Transmittance spectra of lactose and maltose on the quartz sheet and metallic nano-slits structure. The enlarged views of the transmittance spectra of lactose (**c**) and maltose (**d**) on the quartz sheet around their characteristic frequencies of 0.529 and 1.12 THz. (**e**) Transmittance spectra of a mixture of lactose and maltose on the metallic nano-slits structure.

The transmittance spectrum of the metallic nano-slits structure with a slit width of 100 nm in the region of 0.1–1.2 THz are plotted in Fig. 4(a). In this frequency band, the transmittance is higher than 76.9%. Therefore, the region of 0.1–1.2 THz is considered as the working frequency band of this slits structure throughout our study. To evaluate the applicability of this structure to the detection of different saccharides without changing its geometrical parameters, lactose as well as maltose is used as a representative analyte. The transmittance spectra of 200 nm thick layers of lactose and maltose deposited on quartz sheet and on metallic nano-slits are investigated. The results are presented in Fig. 4(b), while Fig. 4(c,d) show the enlarged views of the transmittance spectra for the quartz sheet case around 0.529 THz and 1.12 THz. In the case of quartz sheet, the characteristic frequency of lactose is almost invisible, with an absorption signal strength of only 0.2% (black solid line in Fig. 4(c)). The characteristic absorption of maltose is slightly more obvious than that of lactose, with a signal strength of 0.9%, but it is still weak (red solid line in Fig. 4(d)). In contrast, in the presence of metallic nano-slits, the characteristic absorptions of these two saccharides become significantly more intense. The calculated absorption signal strengths for lactose and maltose are 10.5% and 30.1%, enhancing by 52.5 times and 33.4 times, respectively. It is worth mentioning that the characteristic absorption bands of saccharides in the THz regime are usually not overlapped with each other, thus single absorption peak is sufficient to unambiguously identify a particular saccharide. As a result, fingerprint features of lactose and maltose are easily detected in this case. These results show that this metallic nano-slits structure permits the detection of different analytes without changing its geometrical parameters, as long as the characteristic frequencies of the analytes occur within the working frequency band. Fig. 4(e) shows the possibility of using this nano-slits structure to detect a mixture of lactose and maltose with a thickness of 200 nm. The characteristic absorptions of these two saccharides can be simultaneously observed with the absorption signal strengths of 10.3% and 20.1%, indicating that this proposed structure can also achieve multi-sensing which is very difficult to realize by using the resonant structures.

**Figure 5:**
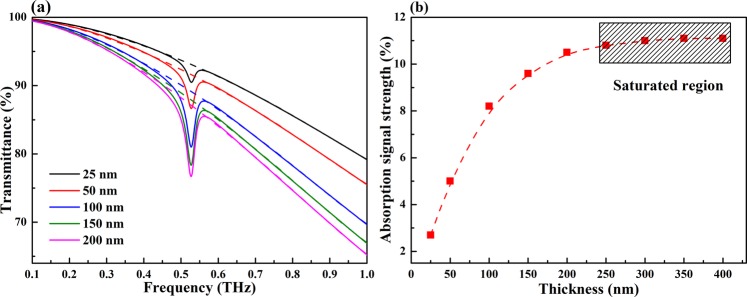
(**a**) Transmittance spectra of layers of lactose with various thicknesses on the metallic nano-slits structure. (**b**) Absorption signal strength as a function of lactose thickness.

The suitability of the metallic nano-slits structure for the quantitative analysis of lactose thickness is also studied. Fig. 5(a) presents the transmittance spectra of layers of lactose with various thicknesses ranging from 25 to 200 nm on the metallic nano-slits structure. The absorption signal strength increases dramatically with increasing lactose thickness. When the thickness reaches 100 nm, the characteristic frequency of lactose becomes readily apparent. Furthermore, the transmittance in the working frequency band decreases significantly with increasing lactose thickness. The absorption signal strength versus lactose thickness is shown in Fig. 5(b). A rapid increase in the absorption signal strength versus lactose thickness is observed when the lactose thickness is less than 100 nm. More specifically, upon increasing the lactose thickness from 25 to 50 nm, the corresponding absorption signal strength increases from 2.7% to 5.0%, and when the lactose thickness is 100 nm, the absorption signal strength reaches 8.2%. A slower rate of increase in the absorption signal strength is observed for lactose thicknesses greater than 100 nm. When the lactose thickness is 150 nm, the absorption signal strength is 9.6%, and a lactose thickness of 200 nm results in an absorption signal strength of 10.5%. However, the absorption signal strength stays almost unchanged and comes into the saturated region when the lactose thickness exceeds 250 nm, shown as the gray shaded area. Therefore, it can be found that when the lactose thickness is thinner than the slit height of 50 nm, the absorption signal intensity nearly linearly increases because the lactose is mostly located inside the slits at which strong and uniform electric fields exist. When the lactose thickness exceeds the slit height, the absorption signal intensity will be enhanced by the gradually decreasing fringe fields, thus leading to saturated absorption signal strength as a function of the lactose thickness. This indicates that the slits structure is very sensitive to thin thickness and is suitable for the detection of trace sample, and the lactose thickness can be predicted on the basis of absorption signal strength when the thickness is less than 250 nm.

## Conclusion

In conclusion, a metallic nano-slits structure has been numerically investigated. The metallic nano-slits structure exhibits high transmittance and electric field enhancement throughout its working frequency band, which facilitates highly sensitive detection of multiple analytes. Here, lactose and maltose are selected as the target analytes, and the calculated results reveal that their absorption signal strengths with a thickness of 200 nm are strongly enhanced by factors of 52.5 and 33.4, respectively. The dependence of absorption signal strength on the lactose thickness variation indicates that the structure is very sensitive to thin thickness and is applicable to the detection of trace sample. The lactose thickness can be predicted on the basis of absorption signal strength when the thickness is less than 250 nm. In contrast to traditional multi-analyte optical biosensors based on resonance matching between the structure and target analyte, the proposed structure can be used to achieve qualitative and quantitative analysis without resonance matching. The purpose of this work is to provide a basis for future experiments. Therefore, expected experimental problems are considered in this work. Although additional problems may be encountered in future experimental studies, we believe that our present theoretical work will provide ideas and methods for solving these problems.
